# Clinical importance of a star shaped branch of internal iliac artery and unusual branches of an abnormal obturator artery: rare vascular variations

**DOI:** 10.1590/1677-5449.000116

**Published:** 2016

**Authors:** Satheesha Badagabettu Nayak, Anitha Guru, Deepthinath Reghunathan, Prasad Alathadi Maloor, Abhinitha Padavinangadi, Swamy Ravindra Shantakumar

**Affiliations:** 1 Manipal University, Melaka Manipal Medical College, Manipal Campus, Madhav Nagar, Manipal, Udupi District, Karnataka State, India.

**Keywords:** iliac artery, obturator artery, inferior gluteal artery, internal pudendal artery, variations, artéria ilíaca, artéria obturatória, artéria glútea inferior, artéria pudenda interna, variações

## Abstract

The internal iliac artery (IIA) is one of the branches of the common iliac artery and supplies the pelvic viscera, the musculoskeletal part of the pelvis, the gluteal region, the medial thigh region and the perineum. During routine cadaveric dissection of a male cadaver for undergraduate Medical students, we observed variation in the course and branching pattern of the left IIA. The artery gave rise to two common trunks and then to the middle rectal artery, inferior vesicle artery and superior vesicle artery. The first, slightly larger, common trunk gave rise to an unnamed artery, the lateral sacral artery and the superior gluteal artery. The second, smaller, common trunk entered the gluteal region through the greater sciatic foramen, below the piriformis muscle and presented a stellate branching pattern deep to the gluteus maximus muscle. Two of the arteries forming the stellate pattern were the internal pudendal artery and the inferior gluteal artery. The other two were muscular branches.

## Resumo

A artéria ilíaca interna (AII) é um dos ramos da artéria ilíaca comum e supre as vísceras da pelve, a parte musculoesquelética da pelve, a região glútea, a região medial da coxa e o períneo. Durante a dissecção de rotina realizada em um cadáver do sexo masculino para estudantes de Medicina, observamos uma variação no curso e padrão de ramificação da AII esquerda. A artéria deu origem a dois troncos comuns e então à artéria retal média, artéria vesical inferior e artéria vesical superior. O primeiro tronco comum, ligeiramente maior, deu origem a uma artéria sem nome, à artéria sacral lateral e à artéria glútea superior. O segundo tronco comum, menor, adentrou a região glútea através do forame ciático maior, abaixo do músculo piriforme, e apresentou um padrão estrelado de ramificação na parte profunda do músculo glúteo máximo. Duas das artérias que formaram o padrão estrelado foram a artéria pudenda interna e a artéria glútea inferior. Os outros dois ramos eram musculares.

## INTRODUCTION

The internal iliac artery (IIA) is one of the two terminal branches of the common iliac artery. The branching takes place at the level of the lumbosacral articular disc and in front of the sacroiliac joint. The artery consists of a trunk and two divisions, namely the anterior division and the posterior division. The arterial trunk passes subperitoneally downwards in front of the sacroiliac joint and, on approaching the upper margin of the greater sciatic foramen, it divides into anterior and posterior divisions.

The anterior division gives off the obliterated umbilical artery, superior vesicle artery, middle rectal artery, obturator artery, uterine artery, vaginal artery, inferior gluteal artery and the internal pudendal artery. The vaginal artery corresponds to the inferior vesicle artery in males. Arising from the posterior division are three branches, the iliolumbar artery, superior gluteal artery and lateral sacral arteries. The branches of the IIA supply the pelvic viscera, the musculoskeletal part of the pelvis, the gluteal region, the medial thigh region and the perineum. The IIA is the proximal part of the umbilical artery whereas its distal end obliterates after birth. Knowledge of the variations in the origin, course and branches of IIA helps in planning and conducting surgeries involving the areas supplied by the artery. Classification of variant patterns of branches of the IIA has been documented.^1^^-^ 5 We report here a rare assemblage of variations involving the IIA and its branches. The surgical and clinical implications of the variations reported are discussed.

## CASE DESCRIPTION

During routine cadaveric dissection of a male cadaver for undergraduate Medical students, we observed variation in the course and branching pattern of the left IIA. The variant vessels were dissected as documented in Cunningham’s manual of practical anatomy.^6^ In this case, the IIA did not divide into anterior and posterior divisions. The artery first gave rise to two common trunks (CT). The first CT, slightly larger than the second, gave rise to an unnamed artery (UNA), to the lateral sacral artery (LSA) and to the superior gluteal artery (SGA). The UNA coursed laterally, superficial to the lumbosacral trunk and supplied the lateral pelvic wall. The inferior gluteal artery (IGA) and internal pudendal artery (IPA) did not arise as separate branches from the internal iliac artery; rather, they arose from the second CT. The second, smaller, CT entered the gluteal region through the greater sciatic foramen, below the piriformis muscle and presented a stellate branching pattern deep to the gluteus maximus muscle. Two of the arteries forming the stellate pattern were the IPA and the IGA. Other branches were unnamed muscular branches (MB). After the second CT, the artery gave rise to the middle rectal artery (MRA), inferior vesicle artery (IVA) and superior vesicle artery (SVA) and then further along its course formed the median umbilical ligament (MUL).

**Figure 1:**
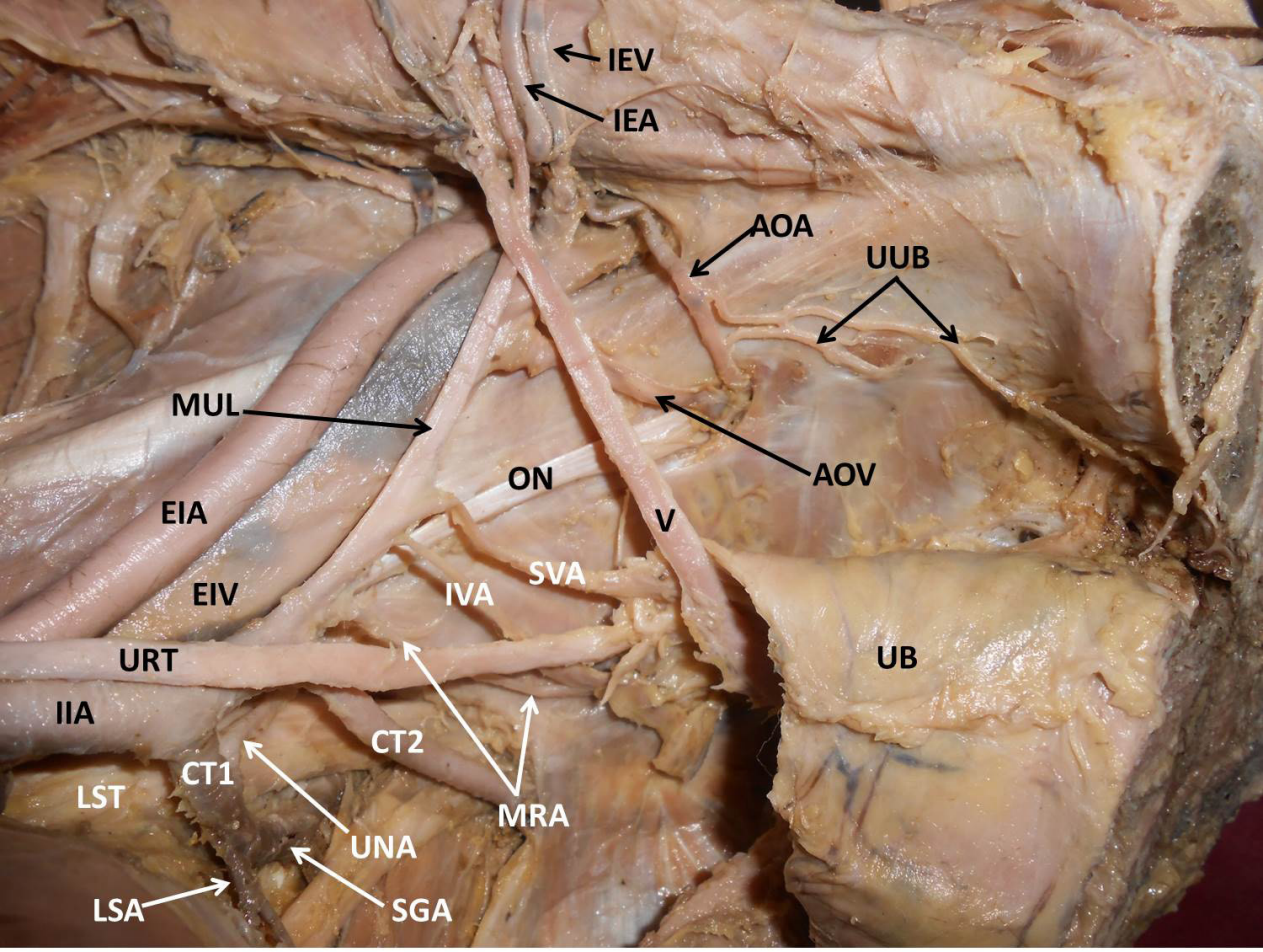
Dissection of the left half of the pelvis showing variant branches of the internal iliac artery. IIA: Internal Iliac Artery; LST: Lumbosacral Trunk; URT: Ureter; EIA: External Iliac Artery; EIV: External Iliac Vein; CT1: Common Trunk 1; UNA: Unnamed Artery; LSA: Lateral Sacral Artery; SGA: Superior Gluteal Artery; CT2: Common Trunk 2; IPA: Internal Pudendal Artery; MRA: Middle Rectal Artery; IVA: Inferior Vesicle Artery; SVA: Superior Vesicle Artery; MUL: Medial Umbilical Ligament; AOA: Abnormal Obturator Artery; AOV: Abnormal Obturator Vein; IEA: Inferior Epigastric Artery; IEV: Inferior Epigastric Vein; UUB: Unusual Branches; UB: Urinary Bladder.

The obturator artery did not arise from the internal iliac artery. Rather, the obturator artery arose from inferior epigastric artery (IEA). It gave off two unusual branches before entering the obturator foramen. These two branches anastomosed with each other on the obturator internus. One of them coursed lateral to the prostate and entered the crus of the penis. The other branch supplied the obturator internus. The obturator vein drained into the external iliac vein. The above variations are shown in Figures 1 - [Fig gf02] 3.

**Figure 2 gf02:**
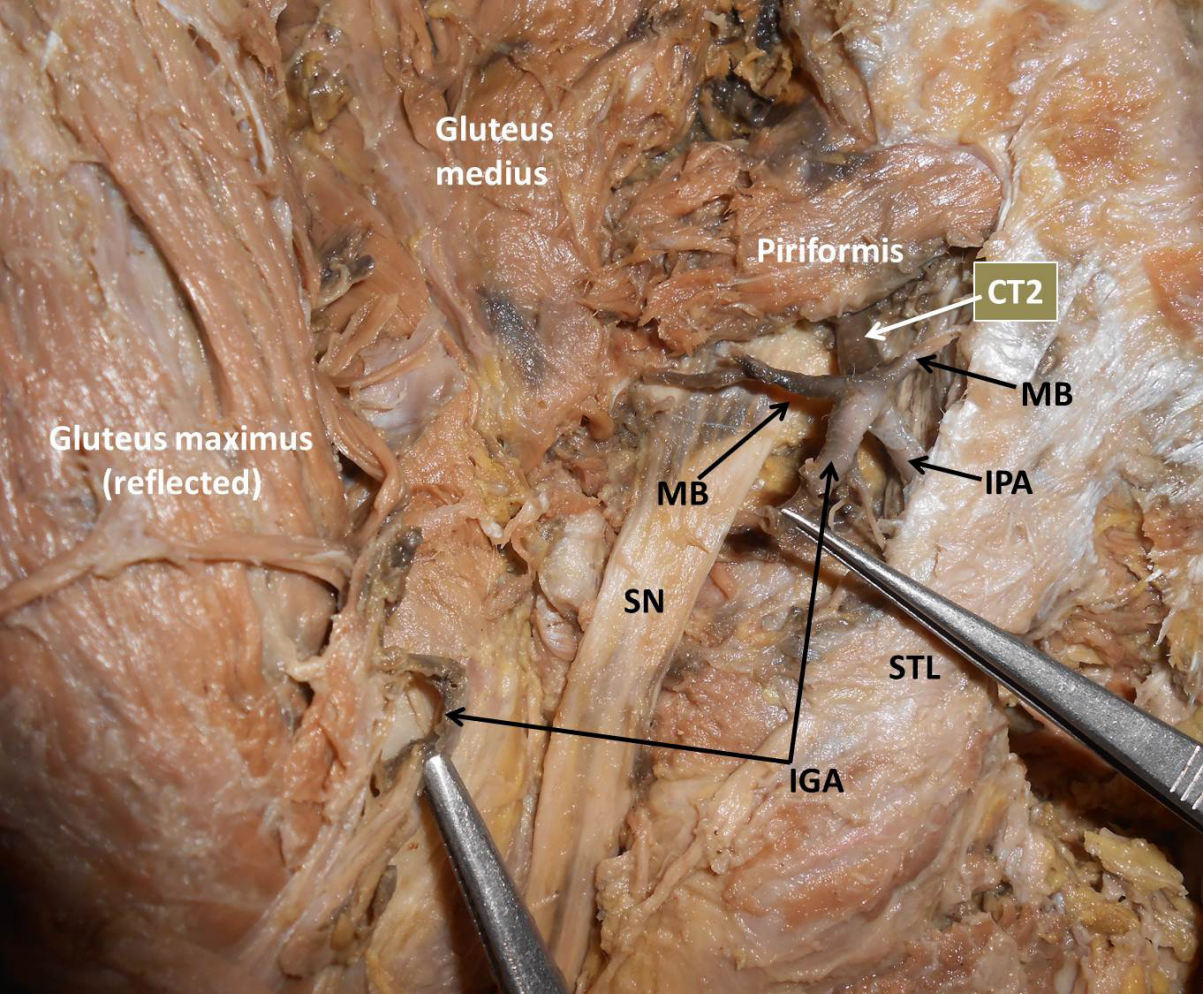
Dissection of the left gluteal region showing variant branches of the internal iliac artery. CT2: Common Trunk 2; MB: Muscular Branches; IGA: Inferior Gluteal Artery; STL: Sacrotuberous Ligament; SN: Sciatic Nerve; IPA: Internal Pudendal Artery.

**Figure 3:**
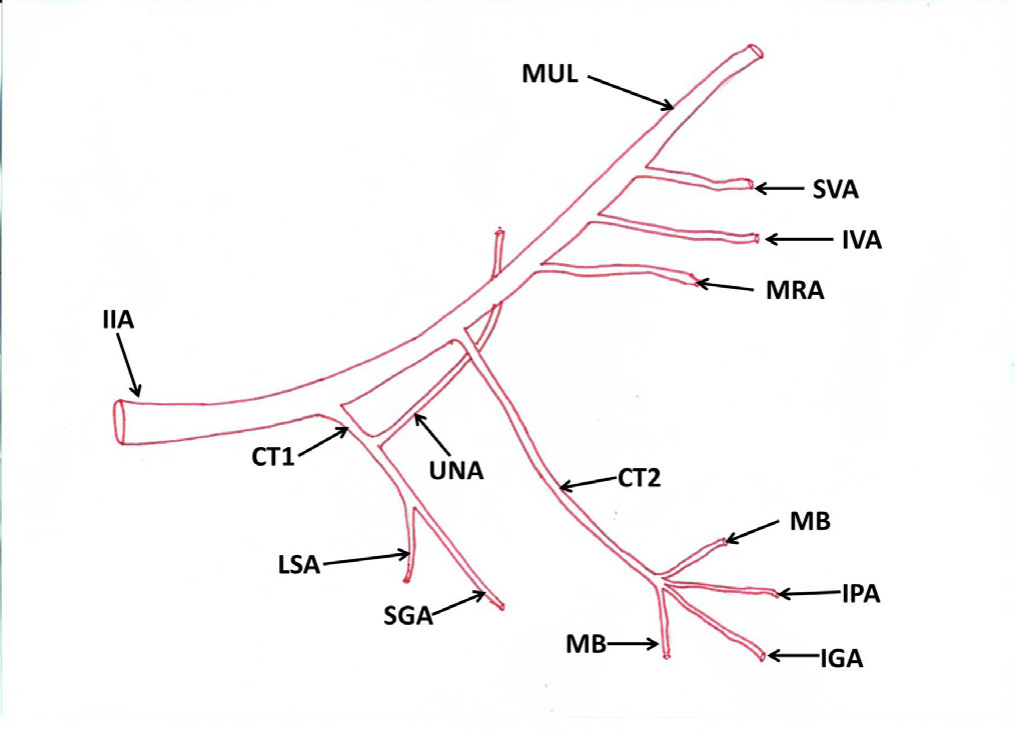
Schematic diagram of variant branches of the internal iliac artery. IIA: Internal Iliac Artery; CT1: Common Trunk 1; UNA: Unnamed Artery; LSA: Lateral Sacral Artery; SGA: Superior Gluteal Artery; CT2: Common Trunk 2; MB: Muscular Branches; IGA: Inferior Gluteal Artery; IPA: Internal Pudendal Artery; MRA: Middle Rectal Artery; IVA: Inferior Vesicle Artery; SVA: Superior Vesicle Artery; MUL: Medial Umbilical Ligament.

## DISCUSSION

**Figure 4:**
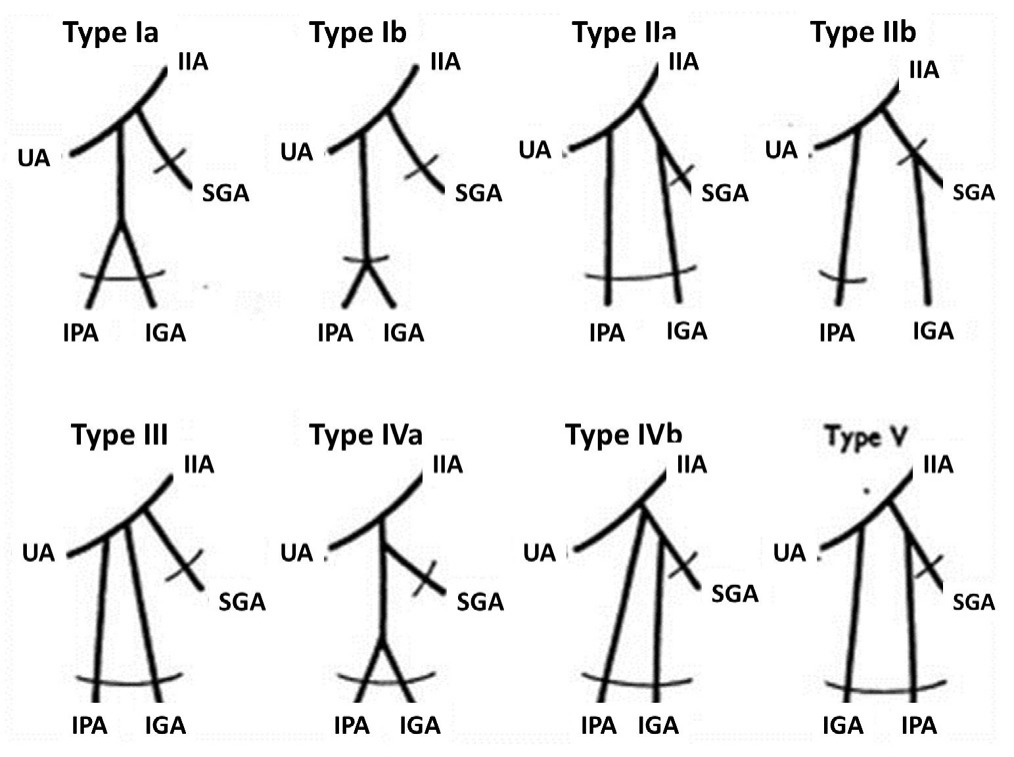
Schematic representation of Adachi’s classification of branching pattern of internal iliac artery. IIA: Internal Iliac Artery; IGA: Inferior Gluteal Artery; IPA: Internal Pudendal Artery; SGA: Superior Gluteal Artery; UA: Umbilical Artery.

Owing to the numerous branches arising from the IIA, variations in the IIA branching pattern have been often reported. In this case, variations were observed in the branching pattern of anterior as well as posterior divisions. For over a century, various authors have devised various classifications of the branching patterns of the internal iliac artery. Way back in 1891, Jastchinski^7^ classified variations of the IIA into 4 types based on a study of variant patterns of internal iliac artery branches in Polish subjects. This classification was modified based on a study of Japanese subjects, including a 5th type of variation and also adding subtypes. Both classifications are based on the origins of the 4 principal branches, namely the umbilical artery, superior gluteal artery, inferior gluteal artery and pudendal artery. Figure 4 shows a schematic representation of Adachi’s types (Braithwaite J.L., 1952).^8^

Following this, a modified classification based on the Adachi classification was proposed by Yamaki et al.^5^ This categorized 5 types of branching pattern and each type had 1 to 6 groups in it. According to this classification, the variation reported falls under type I group 2.

A study of the variability of origin of parietal branches of the IIA stated that the inferior gluteal and internal pudendal vessels were given off by a common trunk in 63.2% of cases.^9^ When the common trunk is divided within the pelvis it is classified as type Ia, which was seen in 60.6%, while the bifurcation occurred below the pelvic floor in 2.6%, i.e., type Ib according to Adachi’s classification. With regard to the posterior division of the IIA, the same study reported variant origins of the superior gluteal artery, together with the iliolumbar artery (2%) or the obturator artery (4%), a variant with double superior gluteal artery (2%), and a variant in which origin was from the anterior division (4%).^9^ In yet another instance of variation, a case of trifurcation of the posterior division of internal iliac artery was reported, in which the posterior division gave rise to the LSA, the SGA and a common trunk.^10^

The uniqueness of the variant described in this case lies in the branching of the IGA and the IPA. The stellate branching pattern, including the common trunk and two muscular branches, was observed in the gluteal region. Branching of the IGA and IPA outside the pelvis has been reported in the literature previoulsy,^1^^,^ 3 ^,^ 5 ^,^ 7 but, to the best of our knowledge, this stellate pattern of branching has not been reported before.

The obturator artery in this case took origin from the inferior epigastric artery, as already reported in the literature. One study has reported a low rate of incidence of the obturator artery originating from the inferior epigastric artery, in 6% of cases,^11^ and in another study it was noted that the obturator artery originated from the inferior epigastric artery in 23.2% of cases in an eastern Indian population.^12^ The obturator artery originating from the inferior epigastric artery has been reported in 25% of cases^3^ and in 19.5% of cases.^13^ However, what makes the current variation exclusive is the origin of the two branches from the obturator artery and their anastomosis, one of them passing to the crus of the penis. These two branches were comparable to accessory pudendal arteries arising from the IIA or any of its branches as reported by other authors.^14^^,^ 15 However, there are no reports of the presence of an anastomosis.

## CLINICAL IMPORTANCE OF THE CASE

During embryonic life, the most appropriate channels of the developing IIA enlarge, while the others disappear, giving rise to a final arterial pattern. In this process, there is a chance of disappearance of one of the major appropriate channels or vice versa, which may result in a variant arterial pattern as reported here. Successful ligation of the IIA is important for surgeons, since efficacy of ligation of this artery in pelvic surgeries varies from 42 to 75%.^16^^,^ 17 An abnormal obturator artery originating from the IEA could be a source of unidentified hemorrhage in pelvic fractures.^18^ An anastomosis between the obturator and the external iliac or inferior epigastric arteries or veins is an anatomical variant termed “corona mortis”. The name “corona mortis” or crown of death testifies to the importance of this feature because significant hemorrhage may occur if it is accidentally cut and it is difficult to achieve hemostasis subsequently. It constitutes a hazard for orthopedic surgeons, especially in the anterior approach to the acetabulum.^19^ Corona mortis, when present, may result in severe bleeding and postoperative complications in fractures of the pubic ramus.^20^ Knowledge of the presence of an artery to the crus of the penis is extremely important because it could result in bleeding during prostatectomy and can also be the cause of erectile dysfunctions.^20^^,^ 21 There are no reports in the literature of such unusual branches of abnormal obturator arteries in females. If present, they could bleed during gynecological procedures.

Knowledge of the stellate branch of the internal iliac artery is of importance to plastic surgeons engaged in raising inferior gluteal artery perforator flaps for breast reconstruction surgery.^22^ The artery could be injured during anterolateral arthroscopy procedures of the hip joint.^23^ Pelvic osteotomy procedures also require a sound knowledge of the variant branches of the internal iliac artery, such as the stellate branch reported here. Such variant branches could bleed profusely during the procedure.^24^ A stellate branch could be mistaken for an inferior gluteal artery pseudoaneurysm in radiological procedures.^25^ The stellate branch is also prone to iatrogenic injuries during surgical procedures in the gluteal region.

## CONCLUSIONS

Though it is common to see variant internal iliac artery branching patterns, the current combination of variant branches including a stellate artery in the gluteal region, an abnormal obturator artery and its unusual branches has not been reported previously. The large stellate artery under the gluteus maximus muscle could cause bleeding during posterior approaches to the hip joint and also in hipbone fractures. Unusual branches of the abnormal obturator artery could get damaged during prostate surgeries. Knowledge of these variations is therefore of importance to general surgeons and orthopedic surgeons.
